# Vocational rehabilitation for adults with psychotic disorders in a Scandinavian welfare society

**DOI:** 10.1186/s12888-016-1183-0

**Published:** 2017-01-17

**Authors:** Erik Falkum, Ole Klungsøyr, June Ullevoldsæter Lystad, Helen Christine Bull, Stig Evensen, Egil W. Martinsen, Svein Friis, Torill Ueland

**Affiliations:** 1 Department of Research and Development, Oslo University Hospital, Oslo, Norway; 2 Institute of Clinical Medicine, University of Oslo, Oslo, Norway; 3 Institute of Psychology, University of Oslo, Oslo, Norway

**Keywords:** Vocational rehabilitation, Psychotic disorders, Barriers to work, Collaboration, Individual support

## Abstract

**Background:**

This study examined the outcomes of a vocational rehabilitation program (The Job Management Program, JUMP) for persons with psychotic disorders based on close collaboration between health and welfare services.

**Methods:**

Participants (*N* = 148) with broad schizophrenia spectrum disorders (age 18–65) were recruited from six counties in Norway. Three counties were randomized to vocational rehabilitation augmented with cognitive behaviour therapy (CBT), while the remaining three counties were randomized to vocational rehabilitation augmented with cognitive remediation (CR). This paper compares the vocational activity of the total group of JUMP participants with a treatment as usual group (*N* = 341), and further examines differences between the two JUMP interventions. Employment status (working/not working) was registered at the time of inclusion and at the end of the intervention period.

**Results:**

The total number of JUMP participants in any kind of vocational activity increased from 17 to 77% during the intervention. Of these, 8% had competitive employment, 36% had work placements in ordinary workplaces with social security benefits as their income, and 33% had sheltered work. The total number of working participants in the TAU group increased from 15.5 to 18.2%. The JUMP group showed significant improvements of positive (*t* = −2.33, *p* = 0.02) and general (*t* = −2.75, *p* = 0.007) symptoms of psychosis. Significant differences between the CBT and CR interventions were not demonstrated.

**Conclusions:**

The study supports existing evidence that the majority of persons with broad schizophrenia spectrum disorders can cope with some kind of work, given that internal and external barriers are reduced. Those who wish to work should be offered vocational rehabilitation.

**Trial registration:**

ClinicalTrials.gov Identifier: NCT01139502. Registered on 6 February 2010.

**Electronic supplementary material:**

The online version of this article (doi:10.1186/s12888-016-1183-0) contains supplementary material, which is available to authorized users.

## Background

Vocational rehabilitation for persons with psychotic disorders has been a challenge in most Western societies. Although 50–70% wish to work, international studies show that only 10–39% manage to obtain and keep a job [1–3]. Norwegian studies indicate that 4–13% of people with schizophrenia are working (S. Evensen et al., 2015 [4]; [5–7]).

Until recently, Norwegian welfare services have been reluctant to include patients with psychotic disorders in vocational rehabilitation programs. These clients have mostly been offered a disability pension without further ceremony. (Stig Evensen et al., 2016 [8]; OECD., 2013 [9]; [10]). Historically, both welfare personnel and clinicians have held the attitude that persons with psychotic disorders are too vulnerable to participate in working life [11, 12]. The above conceptions are still relatively prevalent in Norway, and are likely an important external barrier to employment in this population.

Recent research does however not support the view that persons with severe mental disorders need protection from modern working life (Burns et al., 2009 [13]). Quite the opposite, work is considered an important part of recovery [14], and is closely linked with improved quality of life (Alonso et al., 2009 [15]; [16]). However, many need help to succeed in working life. Fortunately, modern psychosocial treatment methods have contributed to more optimistic views of the developmental potential and functional capacity of persons with severe mental disorders. Cognitive behaviour therapy (CBT) in schizophrenia was originally developed to help the patient cope with positive symptoms (Sensky et al., 2000 [17]; [18]), and has later been adapted to address negative symptoms, such as social withdrawal and apathy. Controlled studies support the efficacy of CBT in schizophrenia [19, 20], and indicate that CBT can increase coping with work demands [21, 22].

Neurocognitive dysfunction is prevalent in schizophrenia ([23]; Lystad et al., 2014) [24] and has a strong impact on different areas of functional outcome (Bowie et al., 2008 [25]; Shamsi et al., 2011) [26] including occupational functioning [3, 27, 28]. Cognitive remediation (CR) has emerged as a treatment that improves both neurocognitive functioning and functional outcome [29]. Some studies also indicate that the combination of cognitive remediation and various vocational rehabilitation programs has a more favourable effect than vocational rehabilitation alone ([30]; McGurk et al., 2009 [14]; [31]). Whereas CBT mainly targets thought *content* (i.e. delusions, negative automatic thoughts), CR mainly targets thought *process* (i.e. concentration, memory, processing speed).

The JUMP (Job Management Program) study is a 10 month trial aiming to improve vocational outcome for persons with psychotic disorders by targeting both external and internal barriers to employment. Competitive employment was the ultimate goal, but both work placement in ordinary workplaces and sheltered work were considered as positive vocational outcomes, compared to unemployment.

The core functions of the Norwegian welfare society are described by the so-called “Nordic model”, which is a social contract with the ambition of ensuring equal access to health and social services for all according to need (Andersen et al., 2007) [32]. The Working Environment Act (Working Environment Act, 2013) is a central part of the model, and regulates working environment, working hours, and employment protection. Employees are entitled to up to one year sick leave, where the employer pays their salary the first two weeks, and can thereafter bill the Norwegian Labour and Welfare Administration (NAV) for sick leave payouts. Termination of employments during this one-year period is not allowed. Due to the risk of increased expenses and low productivity employers are often reluctant to hire individuals with chronic illnesses. To counteract this reluctance the Norwegian community shares risk by offering incentives to employers for providing work placements for up to 24 months. Work placement in ordinary workplaces should be distinguished from sheltered work, based on the fact that the person is actually faced with normal work demands.

Over the last two or three years, the positive findings of several individual placement and support (IPS) studies [33, 34], which consider competitive employment as the only outcome of interest, have promoted more open discussions of the way vocational rehabilitation in Norway is organised, and the IPS approach is currently tried out. However, individuals in work placements also report satisfaction with their job [10], and IPS studies show that a substantial number of participants do not succeed in obtaining competitive work. This indicates that a broader view on work is warranted in terms of positive outcomes for the individual; such as structure, social interaction and quality of life.

Norwegian vocational rehabilitation services are administered by the Labour and Welfare Services (NAV) and have mainly been outsourced to enterprises which offer services in both sheltered workshops and work placement in ordinary workplaces. The JUMP study comprised one third of all Norwegian counties, and was designed within this organisational model.

Employees in NAV and in the rehabilitation agencies often have limited knowledge about psychotic disorders. Symptoms may appear incomprehensible and even frightening [35]. The JUMP study sought to fill this knowledge gap by offering education on characteristics, risk factors, course and prognosis of psychotic disorders to employees both in NAV and the rehabilitation agencies. Furthermore, experienced clinicians supervised the employment specialists in weekly and ad hoc meetings throughout the project period. Thus, while treatment and rehabilitation normally take place at different arenas, the study integrated psychosocial treatment and rehabilitation and considered the vocational context as a particularly potent arena for change.

The illness related barriers (symptoms and cognitive dysfunction) were addressed by applying methods from CBT and CR in the vocational setting. All participants were offered CBT or CR twice a week over 6 months.

The present paper provides a detailed description of the JUMP study, and examines the vocational activity of the whole intervention group compared with a treatment as usual (TAU) group with psychotic disorders. It further investigates whether the symptom level of the JUMP participants developed unfavourably compared to that of the TAU group. The paper also examines the prevalence of competitive versus non-competitive work, the number of working hours per week, and the main sources of income among the JUMP participants at the end of the intervention period. Lastly, the outcomes of vocational rehabilitation augmented with CBT were compared with those of vocational rehabilitation augmented with CR.

## Methods

### Procedure

JUMP is a multisite vocational rehabilitation program for adults with psychotic disorders in Norway. It is a joint venture between NAV, the Norwegian Directorate of Health, and Oslo University Hospital HF. NAV and mental health centres across all nineteen counties of Norway were invited to participate. Eleven counties were interested. Each of them got a number, and six counties were drawn to participate. The first number was randomly allocated to vocational rehabilitation augmented with CBT, the second to vocational rehabilitation augmented with CR, and so forth, until three counties had been drawn to both interventions. The populations in five of the participating counties are quite similar, while the sixth county is a big city (Oslo). Participants received the intervention provided in their catchment area. In each county the project established teams with members from the local NAV department, a local mental health centre, and one or more vocational rehabilitation agencies hired by NAV. The JUMP study was approved by the Regional Committee of Medical Research Ethics and the Norwegian Data Protection Authority. ClinicalTrials.gov Identifier: NCT01139502.

### Participants

#### The JUMP group

Participants were referred from local mental health centres, general practitioners, and from NAV. Self-referral was also possible. All participants provided written informed consent after a complete description of the study. Persons with broad schizophrenia spectrum disorders were recruited. Exclusion criteria were age outside the range of 18–65, head injury with loss of consciousness for more than ten minutes or requiring medical treatment, neurological disorders, IQ below 70, unstable or uncontrolled medical conditions that could interfere with brain function, violent behaviour, severe alcohol and/or drug dependency, and high suicidal risk. The latter three characteristics were assessed with the Health of the Nation Outcome Scale (HoNOS) [36]. For alcohol or substance abuse, violent behaviour and suicidality, a cut-off was set at a score of 3 or more on items 1–3. Participants were required to understand and speak Norwegian to assure valid neurocognitive test performance. The inclusion period was from August 2009 to March 2013.

#### The TAU group

A comparison group was drawn from the database of The Norwegian Centre of Mental Health Research (NORMENT) at Oslo University Hospital. The NORMENT/thematically organised psychosis (TOP) project is an ongoing naturalistic multi-site study on psychotic disorders. Participants are consecutively recruited from in- and outpatient psychiatric units in six major hospitals in the Oslo region, Norway. The current sample is based on the total group of 341 persons recruited for testing between 2002 and 2012, with a DSM-IV diagnosis of schizophrenia, schizoaffective disorder, or psychosis not otherwise specified. The participants consented to research involving health care and better outcome, They also provided written informed consent that anonymized information could be used in studies involving TOP’s collaborators. The current study has been approved by TOP’s International Review Board (IRB) through an amendment. Written informed consent was given after a complete description of the study.

**Fig. 1:**
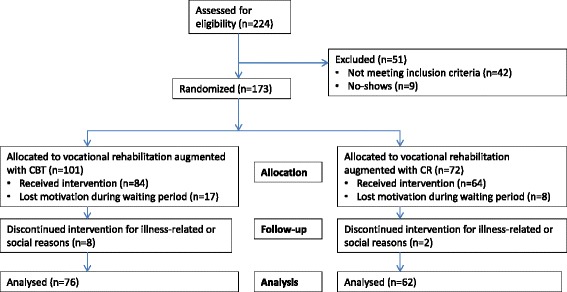
Flow chart

These participants had been assessed at two time points (with a one year interval) with the core instruments applied in the JUMP study, except for the PANSS instrument, which for administrative and logistic reasons had been presented to only 49 of the 341 TAU group participants at the second time point. The baseline PANSS scores of the latter 49 persons were not significantly different from those of the remaining TAU group. Figure 1 shows the participant flow in the two groups.

### Intervention

The JUMP participants were offered ten months of vocational rehabilitation starting with a thorough mapping of the client’s resources and preferences. When an available and suitable job was found either in the vocational rehabilitation enterprise or preferably in an ordinary workplace, the employment specialist offered continuous instrumental and emotional support. All the participants worked actively during the intervention, i.e. the vocational rehabilitation offered differed from so-called prevocational training. Each employment specialist served around ten participants, allowing for close collaboration. The participants had individual CBT or CR sessions with the employment specialist two hours weekly.

The supervision and elements of the education offered to the employment specialists were identical in the two intervention groups. The education component was based on experiences from a previous pilot study, and concerned symptoms, course, treatment, rehabilitation, prevention, and prognosis in schizophrenia and related psychoses [37]. This part of the education was offered to the employment specialists in the participating companies and to NAV employees involved in the study in all counties. Throughout the project period all employment specialists received weekly supervision by an experienced mental health professional, who was also easily available for telephone consultations when problems arose between the meetings.

The employment specialists in the CBT intervention group received training in the basic methods of CBT. The training focused on frequently encountered problems in vocational rehabilitation for persons with psychotic disorders, for instance social withdrawal, apathy, passivity, fear of contact, informal conversation and common meals, drug abuse, and delusions and hallucinations that interfere with work ability. Example: a participant refused to eat lunch in the canteen because she thought her colleagues talked disparaging of her. She isolated herself, and her behaviour made her work mates feel insecure. Helpers without specific knowledge of the vulnerability of persons with psychotic disorders will easily feel helpless confronted with problems like this, whereas employment specialists in the project could use socratic questioning to challenge the participant’s conceptions, actively aiming at cognitive restructuring. Furthermore, they could agree to eat in the canteen together with the participant in order to help her disconfirm her negative belief, a socalled behavioural experiment. Socratic questioning, cognitive restructuring, and behavioural experiments are central CBT concepts. The education made the employment specialists feel secure, and the rather concrete approach made the participants feel taken seriously and respectfully challenged.

In addition, the training addressed basic concepts (such as expressed emotion) and ways of reasoning in the psycho-educative tradition [38, 39]. The training of the employment specialists lasted 40 h.

The employment specialists in the CR intervention group received training about neurocognitive impairment in psychotic disorders, i.e. its characteristics, prevalence and stability, interaction with other symptoms, and consequences for functioning in general and vocational functioning in particular. Furthermore, they were taught the basic principles of cognitive remediation, use of the computer software, strategies to enhance motivation and performance and transfer of knowledge and skills acquired through training to the work setting. The training lasted 40 h and was provided by psychologists with experience in cognitive remediation for patients with psychotic disorders. The CR program included the following elements: Feedback from the neurocognitive assessment, negotiation of personal aims for the training, psycho-education about cognitive impairment, and two hours weekly of computer based training with focus on transfer between training and the work situation. The computer programs targeted attention/vigilance, memory, reasoning and problem solving, and processing speed. The tasks originated from four different programs: COGPACK (Marker Software), Vision Builder (Haraldseth Software), Brain Fitness and InSight (PositScience). Example: A participant had trouble finishing his work tasks. His performance varied greatly without any obvious reason. The neurocognitive assessment and the participant’s performance during the CR sessions indicated that this might result from memory problems. The computer programs targeted memory functions, and the employment specialist helped the participant develop strategies compensating for memory difficulties – both in the CR sessions and on the job. Thus the participant was enabled to try out new cognitive techniques from the training sessions in the vocational setting, and his performance improved substantially.

### Measures

#### Background data

Gender, age, IQ, ethnicity, marital status, education level, previous work experience, and housing were included as descriptive variables in the present paper. Current IQ was estimated with the Wechsler Abbreviated Scale of Intelligence (WASI, 2007), [40, 41] either the two subtests form (applied in the JUMP group) or the four subtests form (applied in the TAU group). Both versions provide a full scale IQ score (FSIQ). Education level was assessed through interviews performed by experienced clinicians. The highest completed level of education was reported. Ethnicity was included as a dichotomous variable (Caucasian/not Caucasian) in the present paper, since between eight and nine out of ten participants were Caucasian in both the JUMP group and the TAU group. Likewise, marital status was recorded as single/not single, and housing as living alone/not living alone on the assumption that these dichotomies are the central ones in our vocational rehabilitation context. Employment history was registered as the total number of months in part time or full time competitive employment or work placement.

#### Clinical assessments

Clinical assessment was carried out by trained clinicians. The Norwegian version of the M.I.N.I PLUS (Sheehan et al., 1998) [42] modules A, C, D, K, L, and M was used for diagnostic purposes in the JUMP group, whereas The Structured Clinical Interview for DSM-IV (SCID 1) (First et al., 1995) [43] was used in the TAU group. All assessors were trained on the use of the diagnostic instruments. During assessment, the Longitudinal, Expert, All Data (LEAD) [44, 45] procedure was applied when necessary, with additional course-relevant data collected from physicians, mental health professionals, care providers, etc. The diagnosis variable in the present study has three categories: Schizophrenia, schizoaffective disorder, and other psychoses. Current levels of psychotic symptoms were rated using the Structural Clinical Interview of the Positive and Negative Syndrome Scale (SCI-PANSS) [46]. The assessments were made by trained raters. Duration of illness (DOI) was recorded as the number of years from the patient’s first contact with specialist health services for psychotic symptoms. The defined daily dose (DDD) of antipsychotics was recorded according to the World Health Organisation [47]. Alcohol and drug use were recorded by clinicians on the 5-point Clinician Rating Scale (Mueser et al., 1995) [48], which was dichotomized (abuse/not abuse) in the present paper.

#### Outcome measures

Employment status (working/not working) among the JUMP participants was registered at the time of inclusion and at the end of the intervention. Work status in the TAU group was registered at two points of time with a one year interval. Both competitive work, work placement in ordinary workplaces, and sheltered work counted as vocational activity. Number of working hours in the JUMP group was measured as hours per week at the end of the intervention. This measure was not available for the TAU group. The same is true for the main sources of income, which are reported for the JUMP group only.

### Analyses

IBM SPSS Statistics version 20.0 and R [49, 50] were used for statistical analyses. All tests were two-tailed, and if not indicated otherwise, Chi Square tests were applied when comparing categorical data, and Student t-tests and analysis of variance (ANOVA) were applied for group comparisons of continuous data. Multiple logistic regressions were used to assess the group difference in positive employment change, adjusted for confounders.

Both in psychiatric and related research fields variables are often contaminated by measurement error, either due to bad assessment tools, because the true variable cannot be measured directly or due to poor inter-rater reliability. Ignoring these issues may lead to serious bias in estimated parameters. The simulation and extrapolation method (SIMEX) ([51]; Lederer, Küchenhoff, Lederer, & by Küchenhoff, 2009) [52] is a useful method to correct effect estimates for additive measurement error. In the present study, the PANSS variable (positive, negative, and general symptoms of psychosis) was expected to be contaminated by measurement error in the JUMP group, and because the participants’ symptom intensity was considered to be an important confounder, the SIMEX method was used to correct the effect estimates in the logistic regression. The T-tests applied for comparison of change in symptom level from T1 to T2 between the JUMP group and the TAU group were also adjusted for additive measurement error (Additional file 1: Appendix 1).

Power calculations indicated that we would need approximately 150 participants in the JUMP group. With a significance level of 0,05 and a power of 0,80, a standardized group difference of 0,44 could be detected between two intervention groups á 80 participants, whereas standardized differences close to 0,80 between supported employment and treatment as usual had previously been found in American studies (Velligan & Gonzales 2007).

The reports from the JUMP study adhere to CONSORT guidelines for reporting clinical trials.

## Results

### Sample characteristics

Table 1 displays the sample characteristics of the JUMP and the TAU groups at T1. Moderate group differences were demonstrated for gender, age, previous work experience, the number of respondents living alone, diagnosis, positive symptoms of psychosis and the use of antipsychotics.

**Table 1:** Sample characteristics (T1)

Variable	Category/measurement unit/scoring ()	JUMP *n* = 148 (percent/Mean/SD)	TAU *n* = 341 (percent/Mean/SD)	Chi^2^/F/df/p
Gender	Male (1)	69.6%	56.6%	Chi^2^ = 7.30/1/< .01
Age	Years	32.88 (7.95)	34.90 (9.39)	F = 5.27/1/p < .05
IQ	WASI	101.55 (13.87)	99.10 (15.96)	F = 2.30/1/ns
Education	Primary school (1)	33.0%	37.1%	Chi^2^ = 5.92/4/ns
	High school (2)	34.6%	29.4%	
	Vocational school (3)	13.7%	9.8%	
	College (4)	10.4%	10.4%	
	University (5)	8.2%	13.4%	
Previous work experience	Months	59.64 (66.43)	76.02 (83.67)	F = 4.20/1/< .05
Marital status	Single (1)	85.8%	80.4%	Chi^2^ = 1.92/1/ns
Housing	Living alone (1)	59.5%	66.6%	Chi^2^ = 0,15/1/<.05
Etnicity	Caucasian (1)	88.5%	83.0%	Chi^2^ = 0.13/1/ns
Diagnosis	Schizophrenia (1)	89.1%	71.3%	Chi^2^ = 18.53/2/< .001
	Schizoaffective (2)	7.5%	17.3%	
	Other psychosis (3)	3.4%	11.4%	
Duration of illness	Years	7.15 (6.37)	7.91 (6.89)	F = 1.25/1/ns
PANSS positive	Sum score	13.15 (4.54)	14.85 (5.66)	F = 9.97/1/< .01
PANSS negative		16.04 (5.65)	15.42 (6.40)	F = 0.99/1/ns
PANSS general		29.30 (8.18)	31.42 (8.80)	F =6.12/1/<.05
PANSS total		58.35 (15.41)	61.80 (17.65)	F = 2.11/1/<.05
Antipsychotics DDD^a^	%/Mean (SD)	95/1.07 (0.8)	86.3/1.26 (1.1)	F = 3.85/1/=.05
Alcohol use	Abuse (1)	8.1%	7.3%	Chi^2^ = 4.15/4/ns
Drug use	Abuse (1)	6.8%	8.5%	Chi^2^ = 8.93/4/ns

*SD* Standard Deviation

^a^ Defined daily Dose (DDD)

### Employment status in the JUMP and TAU groups

Seventeen percent (*n* = 25) of the JUMP participants were working at T1. When the vocational rehabilitation program ended, the total number of participants with any type of work (competitive, work placement at ordinary workplaces or sheltered) had increased to 77% (*n* = 114) of those who started in JUMP (*N* = 148). When the number of included persons (intent to treat, *N* = 173) was applied as denominator, 65.9% of the JUMP participants were working at the end of the intervention period. The corresponding figures in the TAU group (*N* = 341) were 15.5% (*n* = 53) at T1 and 18.2% (*n* = 62) when assessed twelve months later. The changes in the two groups were strongly and significantly different (Chi^2^ = 160.89, *p* < .001, ES (Cohen’s h) = 1.44).

A series of univariate logistic regression analyses showed that age, alcohol and drug abuse, and the symptom intensity measured at baseline were significantly related to positive change in employment status (not working at baseline, working at the end of the intervention period). When these variables were included in a multiple logistic regression analysis, only the PANSS variables remained significant predictors of positive employment change. The PANSS variables were included as confounders in a multivariate logistic regression analysis of the difference between the JUMP and TAU groups. This analysis also included a variable describing the interaction between group and symptom intensity. Adjustment for measurement error in the SCI – PANSS variable was done with SIMEX for all the PANSS scales. Naive and corrected estimates for the PANSS negative scale are presented in Table 2. The average effects of the PANSS scales were somewhat attenuated after adjustment for measurement error.

**Table 2:** Positive change in employment status (not working at T1, working at T2) without (naïve) and with adjustment for measurement error in PANSS negative (reliability = 0.4) by SIMEX. Logistic regression. *N* = 489

Variable	Naïve			Adjusted		
	B	SE	*p*	B	SE	*p*
Group						
TAU	0	Ref		0	Ref	
JUMP	0.86	0.54	0.11	1.14	0.88	0.2
*PANSS negative*	−0.25	0.03	<0.001	−0.25	0.02	<0.001
PANSS negative x group						
TAU	0	Ref		0	Ref	
JUMP	0.23	0.04	<0.001	0.21	0.06	<0.001

A strong group difference in favour of the JUMP group was observed for negative symptoms of psychosis, with increasing odds ratios for increasing symptom levels (effect modification). For a person with an average level of PANSS negative (=15.6) this gives OR = 82.1 (95% CI: 37.1, 181.6). For the person with an average level of PANSS positive (=14,34), the odds ratio was 60.94 (95% CI: 30.4, 122.0). The corresponding figures for general symptoms of psychosis (PANSS general) were OR = 53.89 (95% CI: 26.8, 108.4).

### Symptom level

Table 3 displays the changes of symptom intensity (SCI-PANSS: positive, negative, and general symptoms of psychosis) between T1 and T2.

**Table 3:** Positive, negative, and general symptoms of psychosis (SCI-PANSS) by start (T1) and end of intervention in the JUMP group and by assessment (T1) and reassessment in the TAU group

SCI-PANSS	Group	T1	T2	Change	T/df/p^a^
PANSS positive	JUMP, N	142	119	115	−2.33/115/0.02
	Mean	13.15	11.96	−0.99	
	SD	4.54	5.11	4.58	
	TAU, N	336	49	49	−1.82/48/0.075
	Mean	14.85	13.20	−1.12	
	SD	5.66	6.33	4.32	
PANSS negative	JUMP, N	141	119	115	−1.96/114/0.053
	Mean	16.04	15.17	−1	−1.68/48/0.1
	SD	5.65	5.19	5.53	
	TAU, N	335	49	49	
	Mean	15.42	14	−1.14	
	SD	6.40	5.49	4.77	
PANSS general	JUMP, N	143	119	117	−2.75/116/0.007
	Mean	29.29	27.20	−2.16	
	SD	8.18	8.61	8.51	
	TAU, N	335	48	48	−2.74/47/0.009
	Mean	31.42	29.60	−2.21	
	SD	8.80	10.10	5.58	

^a^test for H_0_: change = 0

The symptom changes in the JUMP and TAU groups are tested separately and a negative change means improvement (Table 3). The JUMP group showed significant improvement for PANSS positive (*t* = −2.33, *p* = 0.02) and both groups showed significant improvement for PANSS general (*t* = −2.75, *p* = 0.007/*t* = −2.74, *p* = 0.009). Correction of measurement error with an assumed reliability for change in PANSS ≤ 0.7 would imply significant improvement of scores in all scales for both groups.

In the JUMP group those who were working at T2 had lower PANSS positive scores than those who did not (*t* = 2.03, *p* = .003), whereas the PANSS negative and PANSS general scores were not significantly different.

### Working hours, workplaces, and sources of income among the JUMP participants

The participants worked on average 10.94 (SD = 9.64) hours per week during the last four weeks of the JUMP intervention.

Table 4 displays the prevalence of competitive and non-competitive work among the JUMP participants who were employed at T1 and T2. Even though the number of participants who had competitive work increased from 2.7% to 8.1%, the majority of the participants had work paid by NAV (work placement). Forty-four percent of the participants worked in ordinary workplaces at T2, whereas the corresponding figure was 8.8% at T1. The main sources of income among the JUMP participants are also shown on Table 4. The number of participants who lived off their own wages increased from none when the project started to 3.6% by the end of the intervention, whereas work assessment allowance and disability pension were the main sources of income for the majority of the participants at both T1 and T2.

**Table 4:** Competitive and non-competitive work among the JUMP participants who were employed at T1 and T2, and main sources of income among the JUMP participants at T1 (*N* =147) and T2 (*N* = 138)

	T1 N (%)	T2 N (%)
Type of work		
Competitive work	4 (2.7)	12 (8.1)
Work placement at ordinary workplaces	9 (6.1)	53 (35.8)
Work placement at sheltered workplaces	7 (4.7)	43 (29.1)
Permanent sheltered work	6 (4.1)	5 (3.4)
Source of income		
Wages	0 (0.0)	5 (3.6)
Work assessment allowance	90 (61.2)	86 (62.3)
Disability pension	52 (35.4)	46 (33.3)
Other	5 (3.4)	1 (0.7)

### The CBT and CR interventions

The number of participants who were working at the end of the intervention period was not significantly different in the CBT and CR groups, neither was the number of participants who had competitive work, work placement in ordinary workplaces, and sheltered work respectively.

## Discussion

The main finding of the present study was that the number of working participants in the JUMP group increased from 17% at baseline to 77% at the end of the intervention period. Among these, 44% worked in ordinary workplaces, either in competitive jobs or in work placement paid by NAV. Over approximately the same time frame the proportion of working participants in the TAU comparison group remained almost constant and low (16-18%).

The differences in vocational activity between the intervention group and the comparison group at the end of the intervention period probably rely on a series of both external and internal factors. When the JUMP study was launched, adults with severe mental illness were most often not offered vocational rehabilitation, but instead got a disability pension shortly after the diagnosis was established,- We lack precise information on how many of the TAU participants were included in some kind of vocational rehabilitation program, but the number was probably very small, reflecting that work for people with severe mental illness has until recently not been a central issue neither in clinical psychiatry nor in the social services in Norway. Originally, the design of the JUMP study included a control group receiving ordinary vocational rehabilitation. We struggled to recruit this group for almost two years, but very few eligible persons were identified. Thus we had to abandon this control group, and concluded that active vocational rehabilitation was normally not offered to patients with schizophrenia spectrum disorders in Norway. This may be considered a finding in the present study.

The described employer reluctance to hire persons with a chronic illness probably partly explains the low number of competitively employed participants. This reluctance is possibly lower in countries with a weaker protection of employees against dismissal.

Economic incentives to employers for providing work placements probably influenced the finding that as many as 36% of the JUMP participants had work placements in ordinary workplaces, a result that may be viewed from different angles. On the one hand it is maintained that placement in ordinary workplaces should be clearly distinguished from sheltered work and viewed as a transition to later competitive employment, based on the fact that the person is actually faced with normal work demands. On the other hand the regulation may be taken to reflect the so-called ‘benefit trap’ (benefits exceed the amount of income the participant could earn by working), which according to the Organisation for Economic Co-operation and Development (OECD) characterises the Scandinavian welfare societies (OECD 2012). The benefit trap is considered to reduce the chance that the participant will be competitively employed. Empirical findings support the benefit trap hypothesis, but more studies and observation time is needed to decide which interpretation is most valid, as methodologically sound vocational rehabilitation of persons with psychotic disorders has not been carried out in Norway until recently.

Only positive, negative, and general symptoms of psychosis, and the interaction between symptom level and group proved to significantly predict positive employment change in the final multiple logistic regression analysis. Low symptom level predicted higher likelihood of vocational activity at the end of the intervention period.

As the labour market may differ between the counties, the level of positive employment change might be expected to differ significantly by county, but this proved not to be the case in the present study.

The effect of the JUMP interventions was modified by symptom level, that is, participants with high symptom scores tended to profit more from the intervention, compared to treatment as usual, than did those with low scores. Some of the recruited participants did not have active symptoms of psychosis, and this result probably indicates that the importance of comprehensive vocational support increases with increasing symptom intensity. It might also reflect that a certain symptom level is required for the CBT and CR methods to be effective.

We found small but significant improvements of symptom level in the JUMP group, indicating that work in itself does not necessarily negatively affect symptom intensity and that many individuals with psychosis can work despite experiencing psychotic symptoms. The result accords with findings in IPS studies (Burns et al., 2009 [13]; [53]). The participants’ problems handling information may have been counteracted by the positive self-evaluation resulting from inclusion and social recognition.

The JUMP participants worked on average eleven hours per week, i.e. a little less than one third of a full time job. The above described reluctance of employers to hire employees with chronic illnesses is based on the concern that they will not be productive enough. However, an economic analysis of vocational services in Norway indicates that if an employee produces the equivalent of 29% of a full time job, the societal accounts will be positive even if a quarter of a million NOK is spent sponsoring the workplace each year (Steen, Legard, Jessen, Niels, & G., 2012) [54].

Vocational rehabilitation programs have thus far mainly addressed the internal barriers to participation in working life, i.e. the development of skills and competence in the client. Support is offered throughout the rehabilitation process in both the IPS and the JUMP programs. The JUMP study further aimed to develop individual competence through the use of CBT and CR in the vocational rehabilitation context. The fact that the CBT- and CR- interventions had similar impacts on employment may indicate that both methods are valuable additions to the continuous support offered by the employment specialists. However, we were not able to disentangle the specific effects of CBT and CR. A thorough qualitative evaluation of the JUMP program [55] indicated that the interventions were adequately performed and mostly highly appreciated by the participants. Furthermore, the CBT and CR educations increased the employment specialists’ perceived professional competence and made them feel secure in their interaction with the participants. We cannot preclude that improved communication skills and support from the employment specialists are as important mediators of change as the specific CBT and CR effects in the present study. On the other hand, recent research indicates that the patient-clinician relationship has a significant, but small independent effect on healthcare outcomes [56]. The increase of working participants in the JUMP group is perhaps too big to be explained by the relationship alone. This speaks in favour of specific CBT and CR effects.

The collaboration between the health and welfare systems was secured through the organisational model. Whereas vocational rehabilitation in Norway is the responsibility of NAV, the IPS model is normally administered by clinical psychiatric teams. The IPS organisation is simpler in the sense that the exchange of knowledge between health and welfare services is not necessary. Such exchange was a core element in the JUMP study, with the overall aim of comprehensive system change. These organisational issues should be thoroughly addressed following the current IPS trials in Norway.

A major strength of the JUMP study is that it integrated treatment and vocational rehabilitation for persons with severe mental disorders. Further, the study addressed both internal and a series of external employment barriers. The study also has several limitations.

To thoroughly examine the independent effects of CBT and CR, an intervention group receiving the comprehensive support only should have been included. Another limitation of the present study is the reduction in number of participants assessed with PANSS at T2 in the TAU group. We cannot guarantee that the TAU patients responding at T2 are representative of the TAU group. However, a bias testing did not reveal significant differences between those who were re-evaluated and those who were not.

The JUMP study involved around 70 co-workers (employment specialists, employees in NAV, and in the mental health centres). Traditionally, multi-centre designs and large numbers of professional co-workers create problems with scientific control. The JUMP study was conducted as an integral part of the vocational rehabilitation practices, and adaptation to organisational and financial frames established for other than scientific purposes was necessary. For instance, the attrition rate would probably have been lower if the participants could have started working without long periods of waiting for a position in the companies. Furthermore, the reliability problems described above may partly have resulted from the high number of raters. Although they were trained in the study, different diagnostic cultures and practices in their local vocational setting may over time have counteracted calibration and contributed to measurement error.

The results of the present study should be interpreted with some caution. The strong organisation of the project has no parallel in the normal health and welfare services in Norway. There is a way to go before the integrated collaboration in the JUMP study describes the general collaboration of the service institutions.

More studies of integrated treatment and vocational rehabilitation are needed. They should apply both qualitative and quantitative methods. Whereas randomized controlled studies are needed to identify specific intervention effects, evaluation studies inform on process factors and may contribute to more precise hypotheses. Both internal and external barriers to employment should be addressed. The external barriers are likely to vary by a series of cultural and basic societal factors. Studies investigating external factors such as prejudice in leaders and colleagues, inflexible job demands, restrictive attitudes among welfare workers, lack of focus on resources and competence among clinicians, and level of collaboration between the health and welfare systems seem particularly important.

## Conclusion

The study indicated that many persons with broad schizophrenia spectrum disorders can work, given that internal and external barriers are reduced. Those who wish to work should be offered vocational rehabilitation.

## Additional file

Additional file 1: BMC Psych Falkum Appendix 1 13 Dec 2016.docx. (DOCX 16 kb)

## Abbreviations

ANOVA: Analysis of variance
CBT: Cognitive behaviour therapy
COGPACK: The neuropsychological cognitive training package
CR: Cognitive remediation
DDD: Defined daily dose
DSM-IV: Diagnostic and statistical manual - iv
HoNOS: Health of the Nation Outcome Scales
IBM SPSS: IBM (International Business Machines) Statistical analysis software package
IQ: Intelligence quotient
JUMP: Job management program
LEAD: Longitudinal, expert, all data
M.I.N.I. PLUS: MINI international neuropsychiatric interview
NAV: The Norwegian Labour and Welfare Administration
TAU: Treatment as usual
NOK: Norwegian kroner
NORMENT: Norwegian Centre of Mental Health Research
OECD: The Organisation for Economic Co-operation and Development
R: R Foundation for Statistical Computing (programming language)
SCID1: Structured Clinical Interview for DSM-IV axis 1 Disorders
SCI-PANSS: Structured Clinical Interview for PANSS (Positive and Negative Syndrome Scale)
SIMEX: The Simulation and extrapolation method
TOP: Thematically organised psychosis project
WASI: Wechsler abbreviated scale of intelligence
WHO: World Health Organisation
