# Supplementary material for: Vocational rehabilitation for adults with psychotic disorders in a Scandinavian welfare society
Source: BMC Psychiatry. 2017 Jan 17;17:24. doi: 10.1186/s12888-016-1183-0 (PMC5240414; doi:10.1186/s12888-016-1183-0)
Supplement: Additional file 1: — BMC Psych Falkum Appendix 1 13 Dec 2016.docx. (DOCX 16 kb) [file 12888_2016_1183_MOESM1_ESM.docx]

**Appendix 1**

In a simple additive measurement error model, the observed value (OBS) can be thought of as the sum of the true value and measurement error (ME), symmetrically distributed around the truth, by OBS=TRUE+ME. The reliability of OBS expresses the proportion of the variance which is contributed by the true value: $reliability={{SD}_{true}^{2}}/\left( {SD}_{true}^{2}+{SD}_{obs}^{2} \right)$. A simple correction for measurement error in the one – sample t – test was done by replacing the observed standard deviation (${SD}_{obs})$ with ${SD}_{true}={SD}_{obs}\times\sqrt{reliability}$ . Since $reliability<1$, this implies lower p – values. Under assumptions of uncorrelated measurement errors from $T_{1}$ to $T_{2}$ and standard deviations of similar magnitude at $T_{1}$ and $T_{2}$, it follows that the reliability of the change variable will be less than the reliability at T_1_ and at T_2_. With a low assumed level of the reliability, the interval between the naive (without correction) and the corrected estimate contains the true parameter value with high probability, and describes the influence of the measurement error.
